# The Effect of Nutritional Supplementation in Ex Vivo Lung Perfusion Perfusate on Human Lung Endothelial Cell Function

**DOI:** 10.3390/cells14211668

**Published:** 2025-10-25

**Authors:** Dejan Bojic, Kimberly Main, Tanroop Aujla, Olivia Hough, Shaf Keshavjee, Mingyao Liu

**Affiliations:** 1 Latner Thoracic Research Laboratories, Toronto General Hospital Research Institute, University Health Network, Toronto, ON M5G 2C4, Canada; danny.bojic@utoronto.ca (D.B.); kim.main@mail.utoronto.ca (K.M.); tanroop.aujla@uhn.ca (T.A.); olivia.hough@uhn.ca (O.H.); shaf.keshavjee@uhn.ca (S.K.); 2 Institute of Medical Science, Temerty Faculty of Medicine, University of Toronto, Toronto, ON M5S 1A8, Canada; 3 Department of Physiology, Temerty Faculty of Medicine, University of Toronto, Toronto, ON M5S 1A8, Canada; 4 Department of Surgery, Temerty Faculty of Medicine, University of Toronto, Toronto, ON M5S 1A8, Canada

**Keywords:** EVLP, nutritional supplementation, perfusate optimization, glutamine, glutathione

## Abstract

Clinical application of ex vivo lung perfusion (EVLP) has increased marginal donor lung utilization. It has been developed as a platform for donor lung reconditioning. However, many of the current repair strategies are limited by a maximum reliable EVLP circuit duration of 12 h. Past studies have successfully extended EVLP through nutrient supplementation, but the exact components and respective mechanisms by which EVLP is extended remains unknown. As such, the focus of this study was to systematically evaluate the effects of nutritional supplements in EVLP perfusates on cell apoptosis, viability, confluence, and migration. To test this, we developed a high-throughput human lung endothelial cell culture platform where experimental perfusates with various combinations of GlutaMAX (a glutamine dipeptide), Travasol (amino acids), Intralipid (lipids), Multi-12 (vitamins), cysteine, and glycine were tested using the Incucyte Live imaging system. GlutaMAX supplementation alone significantly reduced apoptosis, improved viability and cell migration beyond all other supplements and further outperformed standard endothelial cell culture medium. Travasol offered short-term benefits, while Intralipid offered minimal functional support. Multi-12 improved viability and apoptosis independently and in combination with other supplements. The best experimental perfusate targeted the glutathione synthesis pathway, combining GlutaMAX, cysteine and glycine and further reduced apoptosis compared with GlutaMAX alone. Collectively, these results suggest that nutrient selection during EVLP is critical and highlights the need to systematically evaluate perfusate modifications as opposed to broad-spectrum nutrient delivery. This in vitro model provides a cost-effective platform for preclinical screening of perfusate modifications to enhance organ viability during EVLP.

## 1. Introduction

End-stage lung failure is a critical condition that significantly affects patients and healthcare systems worldwide [1,2]. For many patients, treatment options are limited to symptom management, with lung transplantation offering the only curative intervention. However, low organ donation and utilization rates lead to increased morbidity and mortality among transplant waitlisted patients [3,4].

Ex vivo lung perfusion (EVLP) is an innovative technology that enables clinicians to more accurately assess marginal donor lung function by assessing pulmonary mechanics and gas-exchange capacity under normothermic conditions [5,6]. During EVLP, donor lungs are mechanically ventilated and perfused with an acellular perfusate, allowing for continuous physiological and functional evaluation to determine graft suitability for transplantation [6].

Since its introduction, EVLP has significantly increased the number of lungs utilized in transplant [7,8]. Interest in EVLP as a platform to facilitate ex vivo organ repair and reconditioning with the use of pharmacological agents, stem cells, and genetic modification has also grown [9,10,11,12,13]. However, current clinical EVLP protocols are limited by a maximum reliable on-circuit duration of around 12 h [14,15]. Thus, strategies to extend clinical EVLP beyond 12 h are of great interest and necessary to overcome the latency period for translation associated with many therapeutic interventions to facilitate long-term repair [9,10,11,12,13,16].

A very important component of EVLP is the perfusate composition. Many EVLP protocols worldwide use Steen solution (XVIVO, Göteborg, Sweden), an acellular perfusate that uses albumin and dextran 40 to provide oncotic support, electrolytes to prevent vasospasm, and glucose for cellular energy requirements [17]. However, a recent evaluation of the lung metabolome on EVLP suggested that the lack of nutrient diversity may be leading to progressive graft decline [18]. To address this obstacle, several studies have attempted to introduce diverse and targeted nutritional regimens into the EVLP system. Total Parenteral Nutrition (TPN) represents a class of intravenous nutrient formulations designed to support patients unable to meet dietary needs enterally [19]. These solutions can be tailored to include amino acids, lipids, vitamins, and minerals based on the patient’s condition and metabolic demands. Continuous infusion of complete TPN to the EVLP system extended porcine EVLP to 24 h [15,20]. Alternatively, adding a targeted nutrient (L-alanyl-L-glutamine (GlutaMAX^R^, (ThermoFisher Scientific, Mississauga, ON, Canada)) to Steen solution extended EVLP to 30 h [21]. Finally, when both TPN and GlutaMAX were added together during EVLP, the procedure was further extended [22].

While these studies have highlighted the potential benefits of added nutrient support to EVLP, the exact composition, ratio, and concentration of nutrients to support prolonged lung function remains unclear. Performing such studies using whole lung EVLP models would be costly and labour-intensive due to the sheer number of groups that would be required. Thus, in the present study, we developed an in vitro model to systematically investigate the effects of various nutrient combinations and doses on lung endothelial cell confluence, migratory capacity, viability and apoptosis.

Our findings demonstrated that antioxidant-based supplementation was the most effective strategy, promoting superior cellular function to other nutrient based interventions and the conventional un-supplemented perfusate.

## 2. Materials and Methods

### 2.1. Mathematical Modeling

The EVLP circuit is primed with 1.5 L Steen solution. In porcine EVLP studies GlutaMAX was added to the Steen solution at a concentration of 4 mM to prime the system [21,22]. During EVLP, TPN components either alone, or together with GlutaMAX were continuously infused into the perfusate [15,22]. The concentrations of these nutritional components in the perfusate can be initially described as:

Nutrient concentration: y = (Hour of EVLP) × (Infusion rate)(1)

During EVLP, 100 mL perfusate was removed and 100 mL fresh Steen solution was added to the circuit every 2 h, which dilutes the nutritional supplements. Equation (1) was thus adjusted to account for the dilution factor of perfusate replacement:

Nutrient concentration: y = [(Hour of EVLP) × (Infusion rate)]/Dilution Factor(2)

These data were used to select concentrations to be tested in the present study.

### 2.2. Experimental Perfusate Preparation

Nutrient-modified perfusates (referred to as experimental perfusates moving forward) were freshly prepared utilizing Steen solution (XVIVO, Göteborg, Sweden, REF # 19004) as the base. The following nutrient solutions were tested: 100× GlutaMAX (ThermoFisher Scientific, Mississauga, ON, Canada, Catalogue #35050061), Travasol (Baxter Corporation, Mississauga, ON, Canada, DIN 00872296), Intralipid (Fresenius Kabi, Toronto, ON, Canada, DIN 02065673), Multi-12 (SANDOZ Canada Incorporated, Mississauga, ON, Canada, DIN 02100606), and Glycine (Catalogue #G7126) and Cysteine (Catalogue # C7352) (Millipore Sigma, St. Louis, MO, USA). Experimental perfusate electrolyte concentrations and pH were evaluated using a blood gas analyzer (Siemens, RapidPoint 500, Sudbury, United Kingdom).

### 2.3. Cell Culture Screening Model

Human pulmonary microvascular endothelial cells (HPMECs) (donated from Kirkpatrick’s research laboratory) [23] were cultured to sub-confluence in M199 culture medium (ThermoFisher Scientific, Mississauga, ON, Canada, Catalogue #11150059) with 20% fetal bovine serum and 1% antibiotics (Penicillin-Streptomycin) (ThermoFisher Scientific, Mississauga, ON, Canada, Catalogue # 15140122) in a 37 °C 5% CO_2_ incubator. After reaching ~70–80% confluence, cells were trypsinized and seeded in a 96-well (clear wall/clear bottom) microplate (Corning, New York, NY, USA, Catalogue #3596) at a density of 1.2 × 10^4^ cells per well. Cells were then further incubated at 37 °C with 5% CO_2_ until 50% confluence, at which point the culture medium was aspirated, and cells were washed twice with PBS. Then, 100 μL of experimental perfusate was added to each well and cells were transferred to the Incucyte SX5 Live Imaging System (Sartorius, New York, NY, USA), where they were maintained at 37 °C with 5% CO_2_ for 48 h.

### 2.4. Cellular Apoptosis

Cells were prepared as outlined in the *Cell Culture Model* section. Caspase 3/7 substrate dye (Sartorius, New York, NY, USA, Catalogue # 4440) at 1:2500 dilution was added to experimental perfusates prior to incubation with the experimental perfusates. Incucyte scanning settings included whole-well imaging, 4× objective lens, green channel with 300 ms exposure, Corning 3596 96-well plate, with images taken every 2 h. Data analysis on Incucyte Analysis was set as: Top-Hat Segmentation: Radius: 10.00 μm and Threshold: 1.00 GCU. Edge Split On: Edge Sensitivity: 35. Cleanup: Hole Fill: 125.00 μm^2^. *N* = 5 images were selected to train the system using outlined parameters.

### 2.5. Cellular Confluence

Cells were prepared as outlined in the *Cell Culture Model* section. Incucyte scanning settings included whole-well imaging, 4× objective lens, phase contrast image, Corning 3596 96-well plate, with images taken every 2 h. Data analysis on Incucyte Analysis was set with the following parameters: Top-Hat Segmentation: Radius: 10.00 μm and Threshold: 1.00 GCU. Edge Split On: Edge Sensitivity: 35. Cleanup: Hole Fill: 125.00 μm^2^. *N* = 5 images were selected to train the system using outlined parameters.

### 2.6. Cellular Migration

Cells were plated at a density of 2 × 10^4^ cells/well and incubated for 24 h to reach confluence. A 700–800 μm wound was generated in each well by the Incucyte Wound Maker 96-Tool (Sartorius, New York, NY, USA). Incucyte scanning settings included whole well imaging, 4× objective lens, phase contrast image, Corning 3596 96-well plate, with images taken every 2 h. Data analysis on Incucyte Analysis was set with the following parameters: Segmentation Adjustment: 0.8. Cleanup: Hole Fill: 10 μm^2^ and Adjust Size: 2 pixels. Filters: Min Area: 500.00 μm^2^. *N* = 5 images were selected to train the system using outlined parameters.

### 2.7. Cell Viability

Cells were prepared as outlined in the *Cell Culture Model* section with the exception that they were plated in 96-well (black wall/clear bottom) microplate (Corning, New York, NY, USA, Catalogue #4580). After 48 h of incubation, the experimental perfusates were replaced with growth media (M199 + 20% FBS) with AlamarBlue dye (ThermoFisher Scientific, Mississauga, ON, Canada, Catalogue # DAL1025) at a 1:10 ratio. After 2 h incubation at 37 °C, culture plates were transferred to the Cytation microplate reader (BioTek, Winooski, VT, United States) to measure fluorescence intensity. Scanning settings included top reading mode, an excitation wavelength of 560 nm, and emission of 590 nm. Detection method: Fluorescence Intensity. Read Type: Endpoint/Kinetic. Optics Type: Monochromators.

### 2.8. Statistical Analysis

Statistical analysis was performed with GraphPad Prism 9.0 (GraphPad Software, San Diego, CA, United States). For comparison across multiple time points, a Two-way ANOVA was performed. For comparison across groups at one time period, an ordinary One-way ANOVA was performed. All values are represented as mean +/− SEM, with a *p* value < 0.05 to represent statistical significance. All experiments were repeated independently to validate results.

## 3. Results

### 3.1. Identifying Nutrient Concentration for Experimental Perfusates

**Figure 1:**
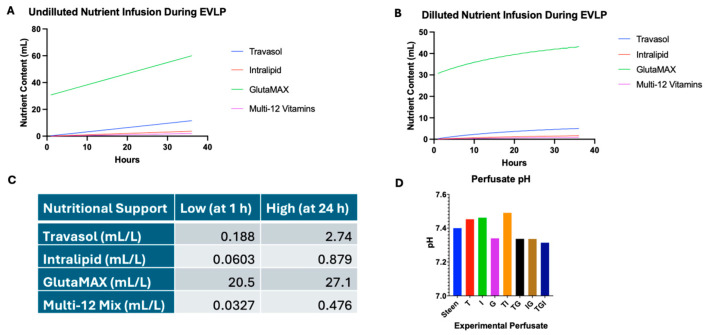
**Determining nutrient supplementation doses for experimental perfusates**. GlutaMAX (G) is used to prime the EVLP circuit, while Travasol (T), Intralipid (I), Vitamins, and additional GlutaMAX are continuously infused during EVLP. To account for dilution from hourly addition of fresh perfusate (100 mL/h), nutrient concentrations were analyzed in both undiluted and estimated diluted conditions. (**A**) Undiluted nutrient input curves show the total amount of each component added to the circuit. (**B**) Diluted curves model estimated in-circuit concentrations over time, accounting for perfusate replacement. (**C**) Estimated nutrient concentrations at 1 and 24 h were used to define low and high doses for downstream in vitro testing. (**D**) The addition of individual or combined nutrient supplements did not significantly alter the pH of experimental perfusates.

To estimate the time-dependent changes in TPN components and GlutaMAX concentrations during EVLP, two equations were developed. The first equation considered the nutrient supplements added to the EVLP perfusate directly, and the second accounted for the periodical removal and addition of Steen perfusate solution throughout EVLP. The expected changes in concentrations of GlutaMAX, Travasol, Intralipids and Multi-12 are plotted in Figure 1A,B, based on Equations (1) and (2), respectively. The total concentration at 1 h and 24 h were selected as low and high concentrations for subsequent in vitro studies (Figure 1C). Evaluation of Steen solution alone revealed a baseline pH of approximately 7.40, and the addition of nutrients at their highest concentrations and in different combinations maintained acceptable ranges in pH (Figure 1D).

### 3.2. GlutaMAX Supplementation Protects Cells from Apoptosis Better than Individual TPN Components

Our nutrient screen started by comparing the effects of solutions that have been previously tested in the context of EVLP. Travasol (a balanced amino acid formulation), Intralipid (a fat emulsion) and GlutaMAX (a stabilized dipeptide of glutamine and alanine) were initially evaluated for their impact on basic cellular functions [15,20,21,22]. Steen solution was used as a baseline control, and M199 culture medium was used as a positive control. In previous studies, EVLP was limited to less than 36 h [15,20,21,22], thus, a 48 h culture period was chosen to determine the limitations of nutrient supplementation.

**Figure 2:**
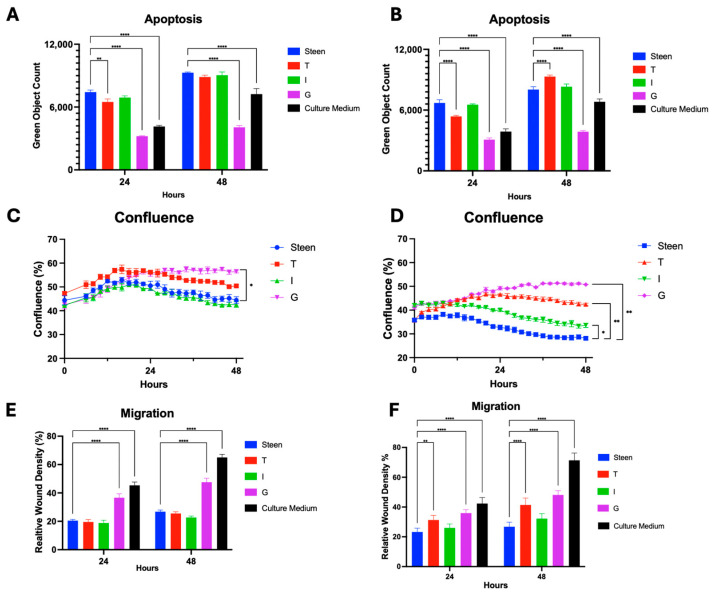
**Effects of individual nutrient supplementation in Steen solution on basic endothelial cell function**. HPMECs were exposed to Steen solution supplemented with individual nutrients for 48 h and monitored using the Incucyte SX5 imaging system. Steen solution and M199 culture medium were used for comparison. (**A**,**B**) Apoptosis at 24 and 48 h was measured under low (**A**) or high (**B**) concentrations. (**C**,**D**) Time course of cell confluence over 48 h under low (**C**) or high (**D**) concentrations. (**E**,**F**) Migration at 24 and 48 h under low (**E**) or high (**F**) concentrations. Statistical analysis: two-way ANOVA, *n* = 3 wells per perfusate. * *p* < 0.05, ** *p* < 0.01, **** *p* < 0.0001.

Lung endothelial cells exposed to Steen solution resulted in increased apoptosis, while M199 significantly reduced apoptosis throughout the experiment. Adding GlutaMAX to Steen solution significantly reduced apoptotic cell count to levels comparable to M199 after 24 h and further reduced apoptosis to levels below M199 after 48 h (Figure 2A,B). Travasol at both low and high concentrations reduced apoptosis only at 24 h, but not at 48 h (Figure 2A,B). In contrast, Intralipid supplementation did not reduce apoptosis at either concentration.

At low nutrient concentrations, endothelial cell confluence gradually increased during the first 16 h and then slightly decreased for all groups except for the GlutaMAX treated group, which was significantly higher than Steen solution at 48 h (Figure 2C). Under high-concentration conditions, GlutaMAX, Travasol, and Intralipid groups all showed significantly higher confluence than Steen solution throughout the entire 48 h. Again, even at high concentrations, GlutaMAX was the most effective supplement in improving cell confluence (Figure 2D).

When cell migration was compared, GlutaMAX supplementation significantly improved cell migration under both low and high concentrations (Figure 2E,F). On the other hand, Travasol showed improvement in migration only at higher concentrations, and Intralipid did not affect migration (Figure 2E,F).

**Figure 3:**
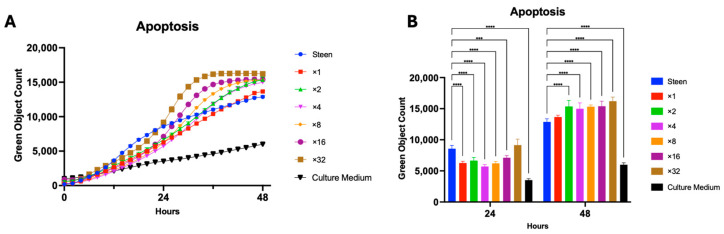
**Dose-dependent effects of Travasol supplementation on endothelial cell apoptosis**. (**A**) Time course of apoptosis over 48 h in cells exposed to increasing concentrations of Travasol. (**B**) Apoptosis at 24 and 48 h of incubation. Travasol concentrations were increased by doubling the high dose used in initial screening experiments. Steen solution and M199 culture medium were included for comparison. Statistical analysis: two-way ANOVA, *n* = 6 wells per perfusate. *** *p* < 0.001, **** *p* < 0.0001.

The functional benefits of Travasol varied in impact between the low and high concentration interventions (Figure 1A,C). This prompted us to a dose escalation study to better identify the relationship between increasing levels of amino acid supplementation and functional outcomes. As such, Travasol concentration was increased from the initial high dose up to ×32 the initial high dose that was tested. Interestingly, during the first 24 h, the apoptosis levels in all Travasol groups (except ×32) were lower than those of the Steen group. Beyond 24 h, apoptosis increased and was significantly higher in all groups (except ×1) compared to the Steen group (Figure 3).

### 3.3. Combined Nutrient Experimental Perfusates Do Not Outperform GlutaMAX Supplemented Perfusate

The second phase of screening experimental perfusates investigated whether combining different nutritional supports could produce synergistic benefits over a 48 h period. To test this, GlutaMAX, Travasol, and Intralipid were combined at low and high concentrations to create multi-nutrient experimental perfusates.

**Figure 4:**
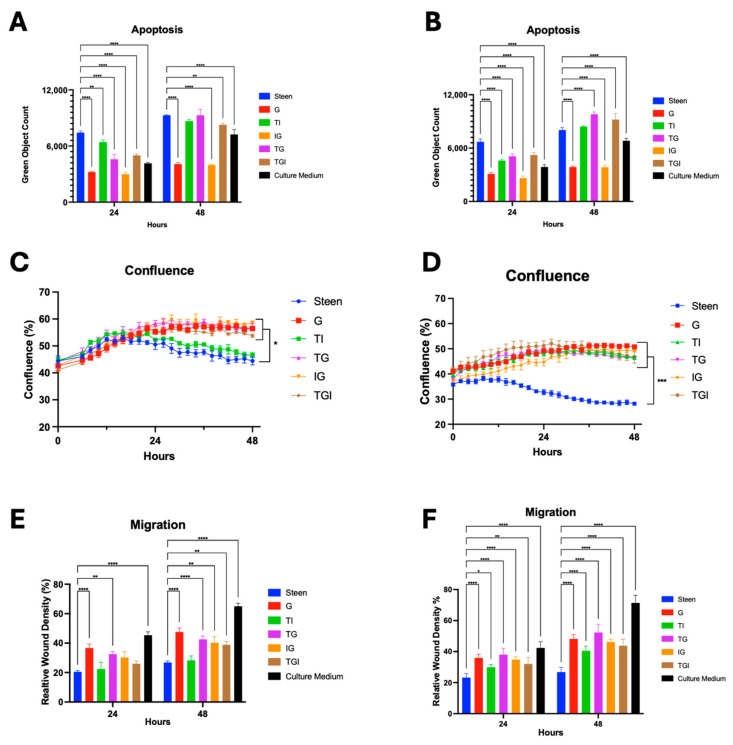
**Effects of combined nutrient supplementation in Steen solution on basic endothelial cell function**. (**A**,**B**) Apoptosis at 24 and 48 h under low (**A**) or high (**B**) concentrations. (**C**,**D**) Time course of cell confluence over 48 h under low (**C**) and high (**D**) concentrations. (**E**,**F**) Migration at 24 and 48 h under low (**E**) and high (**F**) concentrations. Statistical analysis: two-way ANOVA, *n* = 3 wells per perfusate. * *p* < 0.05, ** *p* < 0.01, *** *p* < 0.001, **** *p* < 0.0001.

When Travasol was combined with either GlutaMAX (TG) or Intralipid (TI), a significant reduction in apoptosis was observed at 24 h, but not at 48 h (Figure 4A,B). Combining Intralipid with GlutaMAX (IG) produced a sustained reduction in apoptosis over 48 h, comparable to that of GlutaMAX (G) alone (Figure 4A,B). Perfusates containing all three nutrients (TGI) significantly reduced apoptosis at 24 h at both concentration levels, but not at 48 h (Figure 4A,B).

Under low-concentration conditions, cell confluence and migration were significantly improved in perfusates containing GlutaMAX (TG, IG, TGI) compared to Steen solution at 48 h. Alternatively, the experimental perfusate containing Travasol and Intralipid (TI) did not improve confluence and migration (Figure 4C,E). At higher nutrient concentrations, all experimental perfusates significantly improved both confluence and migration at 24 and 48 h (Figure 4D,F).

### 3.4. Vitamin Supplementation Improves Basic Cell Function for 48 h

**Figure 5:**
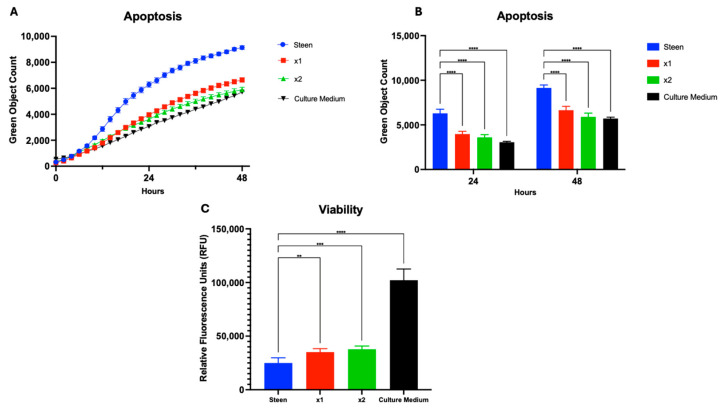
**Multi-12 supplementation reduces apoptosis and enhances cell viability**. (**A**) Time course of apoptosis over 48 h in cells treated with increasing concentrations of Multi-12. (**B**) Apoptosis measured at 24 and 48 h. (**C**) Viability assessment at 48 h using AlamarBlue. Statistical analysis: two-way ANOVA, *n* = 3 wells per perfusate. ** *p* < 0.01, *** *p* < 0.001, **** *p* < 0.0001.

In addition to evaluating macronutrient-based interventions, we investigated the potential utility of micronutrient supplementation in the EVLP perfusate. Multi-12, a vitamin mixture commonly administered to patients with Travasol, was selected for this purpose. We tested increasing concentrations of Multi-12 in experimental perfusates, corresponding to the concentration typically paired with high-dose Travasol (×1) and double that amount (×2) (Figure 1C). Both doses significantly reduced apoptosis and improved cellular viability, reaching levels comparable to those observed with culture medium (Figure 5).

**Figure 6:**
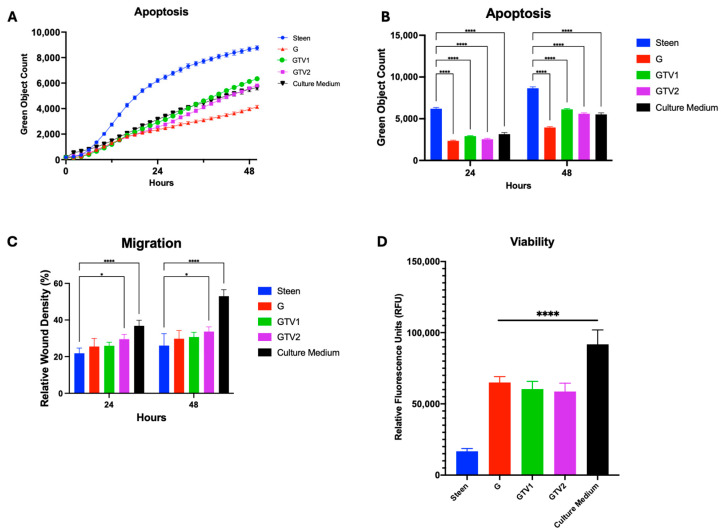
**GlutaMAX, Travasol, and Multi-12 (GTV) combination reduces apoptosis and enhances cell viability**. (**A**) Time course of apoptosis over 48 h. (**B**) Apoptosis measured at 24 and 48 h. (**C**) Migration at 24 and 48 h. (**D**) Viability assessment at 48 h. TGV1 and TGV2 represent combinations at low and high nutrient concentrations, respectively. GlutaMAX (G) was included for comparison. Statistical analysis: two-way ANOVA, *n* = 3 wells per perfusate. * *p* < 0.05, **** *p* < 0.0001.

Building on this, we assessed whether combining all three nutritional supports, which showed individual benefit (GlutaMAX, G; Travasol, T; and Multi-12, V), could provide additive or synergistic benefits beyond those achieved with GlutaMAX alone. These components were combined at two dosing levels: GTV1 (low) and GTV2 (high) (Figure 1C). Both combinations significantly reduced apoptosis (Figure 6A,B) and improved cell viability compared to Steen solution, but not compared to GlutaMax alone (Figure 6D). Additionally, the higher concentration group (GTV2) improved cell migration compared to Steen solution (Figure 6C). Despite these improvements, none of the combined experimental perfusates outperformed GlutaMAX alone in any functional parameter, and GlutaMAX remained the most effective single additive.

### 3.5. GlutaMAX, Cysteine, and Glycine Supplementation Synergistically Reduces Apoptosis and Improves Viability

The finding that GlutaMAX is the most effective nutritional supplement, even when compared to broad-spectrum nutrient regimens, is interesting. While the exact mechanisms underlying its protective effects remain to be further determined, adding GlutaMAX in Steen solution has been shown to increase the biosynthesis of the antioxidant glutathione (GSH) [21]. We proposed that enhancing GSH synthesis by supplementing other key precursor amino acids (cysteine and glycine) might yield further protection beyond GlutaMAX alone.

**Figure 7:**
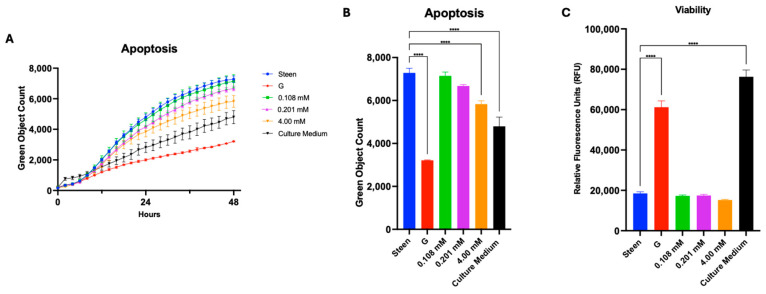
**Effects of cysteine supplementation on basic endothelial cell function**. (**A**) Time course of apoptosis. (**B**) Apoptosis at 48 h. (**C**) Viability at 48 h. GlutaMAX (G) was included for comparison. Statistical analysis: two-way ANOVA, *n* = 3 wells per perfusate. **** *p* < 0.0001.

To test this, cysteine and glycine were added to Steen solution individually or together with GlutaMAX. Dosing concentrations were selected based on levels used in commercially available cell culture media (M199 or DMEM) and based on previous GlutaMAX studies for perfusate modifications [21]. Supplementation with increasing concentrations of cysteine led to a dose-dependent reduction in apoptosis over time (Figure 7A); however, at the end of 48 h, only the highest dose (4 mM) reached statistical significance (Figure 7B). None of the groups improved cell viability, compared with Steen solution (Figure 7C).

**Figure 8:**
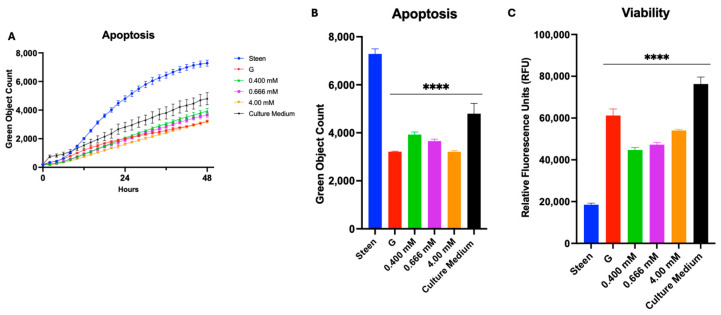
**Glycine supplementation decreases apoptosis and increases viability**. (**A**) Time course of apoptosis. (**B**) Apoptosis at 48 h. (**C**) Viability at 48 h. GlutaMAX (G) was included for comparison. Statistical analysis: two-way ANOVA, *n* = 3 wells per perfusate. **** *p* < 0.0001, each group vs. Steen solution.

Glycine alone at all 3 doses tested reduced apoptosis over that observed with GlutaMAX (Figure 8A,B). Cell viability, measured via the AlamarBlue Assay, was also significantly improved in perfusates containing different doses of glycine (Figure 8C).

**Figure 9:**
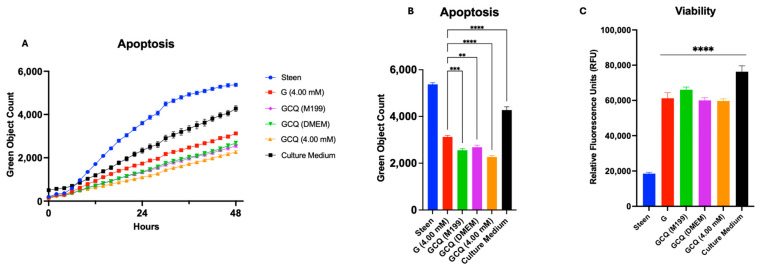
**Glycine, Cysteine, and GlutaMAX (GCQ) supplementation improves basic cell health beyond GlutaMAX alone**. (**A**) Time course of apoptosis. (**B**) Apoptosis at 48 h. (**C**) Viability at 48 h. Experimental perfusates were created to match concentrations found in culture media (M199, and DMEM), and 4 mM for each of them. GlutaMAX (Q) alone was included for comparison. Statistical analysis: two-way ANOVA, *n* = 3 wells per perfusate. ** *p* < 0.01, *** *p* < 0.001, **** *p* < 0.0001.

We then combined glycine, cysteine, and GlutaMAX based on the concentrations used in culture media (M199 and DMEM), and all at 4 mM. Interestingly, all 3 experimental perfusates produced a significant dose-dependent decrease in apoptosis below that seen in the GlutaMAX alone group (Figure 9A). All three combinations (M199, DMEM, and 4 mM concentrations) significantly reduced apoptosis compared to Steen solution and GlutaMAX alone at 48 h (Figure 9B). However, cell viability only improved compared to Steen solution and not GlutaMAX alone at 48 h (Figure 9C). Taken together, these results suggest that targeted nutrient supplementation with GSH substrates offers a promising strategy to reduce apoptosis and may improve Steen solution to extend acellular EVLP over prolonged perfusion periods.

## 4. Discussion

Adding various nutrient supplements to the commonly used Steen solution extended EVLP periods in porcine models [15,20,21,22]. In the present study, we examined the effects of these nutrient supplements on basic cellular function, with the goal of understanding the optimal doses and combinations at which these nutrients may enhance cell function.

### 4.1. Cell Culture Model for Developing New EVLP Perfusates

Many of the advancements in EVLP have relied on porcine lung testing. These models provide a comprehensive assessment of a particular intervention and offer insights for clinical translation. However, these studies are costly, resource-intensive, labor-intensive, and usually focus on evaluating one modification at a time, which significantly restricts the ability to screen multiple potential interventions efficiently.

In this study, we used cell culture-based testing to evaluate perfusate modifications. Using human pulmonary endothelial cells, the primary cell type exposed to the perfusate during clinical EVLP, we tested over 50 individual and combined perfusate modifications. From the perfusates tested, those which showed promise during the initial screening were further evaluated through viability assessments to better characterize the cellular impact of nutrient supplementation. We demonstrated that exposure to the commonly used Steen solution induced apoptosis significantly above nutrient-rich cell culture medium. Of the individual nutrient based experimental perfusates we initially tested, GlutaMAX alone was the most effective at reducing apoptosis, maintaining cell confluence, and promoting cell migration. These improvements exceeded Travasol and/or Intralipid (components of TPN). Moreover, when combining GlutaMAX with TPN components, there are limited additive benefits. These parallel previous observations from porcine EVLP studies [15,20,21,22].

This system represents a promising platform for preclinical evaluation of EVLP perfusate additives through rapid, cost-effective, and evidence-based testing. Importantly, our proposed pipeline offers a path forward for screening interventions beyond nutritional support, potentially informing future directions in perfusate modifications. By narrowing the field of modifications before animal testing, this approach can help reduce unnecessary animal experimentation and promote more targeted translational research.

### 4.2. TPN-Based Supplementation to EVLP Perfusates

Clinically, TPN has been used for patients who are unable to meet nutritional needs enterally, with different brands and combinations [19,24]. Previous research has shown that the addition of TPN to EVLP can extend circuit times [15,20,22]. However, the contribution of each nutritional component was unknown. In the present study, Travasol supplemented experimental perfusates produced transient cytoprotective effects in human lung endothelial cells, with significant improvements in apoptosis, migration, and confluence sustained for up to 24 h (Figure 2). However, beyond this time point, the benefits diminished. These results support previous observations from porcine EVLP studies [15,20], and support the translational relevance of our cell culture studies. High doses of Travasol were associated with increased apoptosis and reduced cell viability, suggesting a threshold at which amino acid supplementation becomes detrimental (Figure 2A,B and Figure 3). At very high concentrations, Travasol increases osmolarity of the solution, which may partially contribute to the increased apoptosis.

Our results show that Intralipid alone did not enhance endothelial cell function compared to Steen solution, except for improved confluence at high concentrations. When combined with other nutrients, no additive benefits were observed. In contrast, Multi-12 alone reduced apoptosis and improved cell viability (Figure 5). When combined with GlutaMAX and Travasol at higher concentration, significant improvement of cell migration was seen (Figure 6).

Taken together, the type of amino acids, lipids, vitamins, and trace elements supplemented should be carefully selected and tested in different combinations when nutritional supplementation is considered as a strategy to enhance EVLP or other ex vivo organ perfusion systems. These considerations should account for both the benefits and potential negatives brought on by nutritional supplementation during EVLP. Moreover, priming the circuit with nutrients before EVLP should be considered, as it could provide relatively stable concentrations of different nutrient supplements rather than a gradual increase. This approach may lead to a more immediate nutrient availability following lung preservation, rather than a delay to reaching optimal circuit concentrations with continuous infusion.

### 4.3. GSH Nutritional Supplementation of Perfusates Offers Better Support

Among all nutritional the supplements evaluated in the present study, GlutaMAX supplemented perfusates consistently outperformed other formulations and even outperformed culture medium, M199. The concentrations of GlutaMAX we used were at least 4 mM (584 mg/L), while in the M199 medium, the concentration of glutamine was much lower at 100 mg/L. Although, Travasol offers a combination of many amino acids, it lacks cysteine, glutamine, asparagine, glutamate, and aspartate. Of interest, critical amino acids that are missing from Travasol are related to GSH biosynthesis. Overall, the lower levels of glutamine in M199 culture medium and the lack of critical amino acids in Travasol may explain why they are not as effective as GSH targeted perfusates.

To further explore the protective mechanism of GlutaMAX, we investigated whether its benefit could be amplified by supporting substrates of GSH. Individually, glycine reduced apoptosis and improved viability (Figure 8), likely due to its favorable transport properties and established role in oxidative stress mitigation [25]. Cysteine, while less effective alone, demonstrated a dose-dependent reduction in apoptosis (Figure 7). When all three components were combined, we observed a significant reduction in apoptosis relative to GlutaMAX alone (Figure 9), supporting the hypothesis that enhancing the GSH pathway contributes to improved cellular resilience under preservation stress. These results align with previous work showing that GlutaMAX increases total GSH levels in the context of EVLP [21]. While GSH can be directly added to a solution, reduced GSH is unstable and its concentration in organ preservation solutions decreases in a temperature- and time-dependent manner through auto-oxidation [26,27]. Thus, delivering the precursors of GSH may be a more practical, effective and physiologically compatible strategy.

Interestingly, supplementing GlutaMAX, cysteine, and glycine together (GCQ groups, Figure 9C) did not further improve cellular viability compared to GlutaMAX alone. This finding suggests that the additional protective effects of GlutaMAX may be limited to oxidative stress mitigation, rather than enhanced proliferation, which is primarily driven by glutamine via mTORC1 activation [28].

The fact that M199 treated cells showed better cell viability than that of GlutaMAX or GCQ groups suggests that the composition of culture medium can promote superior cell survival and proliferation in vitro. One must consider that cell culture media do not possess the oncotic properties to effectively support whole organ perfusion. Proof of concept studies have tested cell culture medium based perfusate wherein dextran 40 and albumin (basic components of Steen solution) were added to DMEM (with GlutaMAX) culture medium as a potential EVLP perfusate. This solution protected cells, promoted cell migration and reduced injury and death after cold preservation during simulated EVLP [29]. Studies utilizing culture medium based perfusates were recently reported upon where DMEM with bovine serum albumin supported porcine lungs on EVLP for 6 h [30]. Such recent studies underscore a growing interest in nutritionally supplemented perfusates that may outperform the current acellular standard perfusates.

These findings demonstrate that targeted nutrient supplementation, specifically through the GSH axis, can significantly improve endothelial cell survival in conditions simulating prolonged EVLP. Further, these results point to a promising direction for next generation perfusate development focused on enhancing nutrition, antioxidant defenses, and cellular resilience, in the ex vivo perfused organ.

### 4.4. Limitations

Our study utilized a cellular based approach to recapitulate the EVLP related condition, which brings several limitations. Primarily, we utilized a monolayer, single cell type culture model. This approach limited our ability to evaluate the impact of experimental perfusates on the diverse cellular microenvironment of the lungs which include endothelial, epithelial, immune, and muscle cells in three-dimensional tissue structure. Moreover, we did not evaluate the inherent stressor involved in the transplant process which include cold ischemia time, warm reperfusion, mechanical ventilation and sheer stress associated with pulmonary vasculature. Further, the intracellular GSH, redox status (GSH/GSSG), reactive oxygen species, lipid peroxidation markers, or GSH synthesis enzyme expression were not measured. Finally, this study did not evaluate injured cells. In the context of EVLP, where there is the assessment of marginal lungs and an outlook for reconditioning, introducing injured cell models may be of particular importance to evaluate the role of nutrient supplemented perfusates.

While our study does not address these important translational factors, it still nonetheless provides an appropriate initial evaluation of potential innovations to the perfusate that may be favourable in the clinical context. Further, our results are able to recapitulate many of the large animal model findings, i.e., TPN based interventions offering benefit for only about 24 h, which further strengthens this approach as an initial screening platform. Future studies should utilize the information from this study to guide evidence-based research practices in more complex animal EVLP models that are able to closely replicate the clinical EVLP process.

## 5. Conclusions

We developed a high-throughput cell screening platform with human endothelial cell culture that systematically evaluated more than 50 perfusate formulations. Targeted supplementation with GSH precursors (GlutaMAX, cysteine, glycine) significantly reduced apoptosis and improved cell viability compared to Steen solution and broad-spectrum nutritional approaches. These findings, consistent with previous porcine studies, identify promising candidates that warrant validation in whole-organ models during EVLP.

## Data Availability

No new data were created or analyzed in this study. Data sharing is not applicable to this article.
